# Impact of Behavioral and Psychological Symptoms on Caregiver Burden in Patients With Dementia With Lewy Bodies

**DOI:** 10.3389/fpsyt.2021.753864

**Published:** 2021-10-27

**Authors:** Hideki Kanemoto, Shunsuke Sato, Yuto Satake, Fuyuki Koizumi, Daiki Taomoto, Atsushi Kanda, Tamiki Wada, Kenji Yoshiyama, Manabu Ikeda

**Affiliations:** Department of Psychiatry, Osaka University Graduate School of Medicine, Suita, Japan

**Keywords:** behavioral and psychological symptoms of dementia (BPSD), dementia with Lewy bodies (DLB), caregiver burden, instrumental activities of daily living (IADL), wakefulness

## Abstract

**Background/Objective:** Behavioral and psychological symptoms of dementia (BPSD) have been reported to affect caregiver burden in patients with dementia with Lewy bodies (DLB). However, the factor structure of BPSD and the factors that affect caregiver burden in DLB remain unknown. This study sought to classify BPSD and to reveal what type of BPSD affects caregiver burden in patients with DLB.

**Methods:** We collected data on neuropsychiatric inventory-plus (NPI-plus), Zarit Burden Interview (ZBI), Mini-Mental State Examination (MMSE), Lawton's Instrumental Activities of Daily Living and Physical Self-Maintenance Scale (IADL/PSMS), age, and sex of 102 patients with probable DLB. An exploratory factor analysis of 13 items of the NPI-plus was carried out to classify BPSD. Multivariate regression analyses were conducted to extract the clinical variables related to caregiver burden, including factors resulting from the aforementioned factor analysis.

**Results:** The mean age and MMSE score were 78.6 (5.6) and 20.2 (5.2), respectively. Factor analysis revealed four factors of “psychosis,” “affection,” “wakefulness,” and “hyperactivity.” “Psychosis” and “affection” factors as well as MMSE, IADL, and PSMS were significantly associated with ZBI. Multivariate regression analyses revealed that the total score of ZBI was associated with “psychosis,” “affection,” and IADL, that the personal strain score of ZBI was associated with “affection” and IADL, and that the role strain score of ZBI was associated with “wakefulness” and IADL.

**Conclusions:** BPSD in DLB consists of three factors common to Alzheimer's disease and a specific “wakefulness” factor. In addition to IADL, each BPSD factor would affect caregiver burden in different ways in DLB.

## Introduction

Dementia with Lewy bodies (DLB) represents the second most common type of neurodegenerative dementia after Alzheimer's disease dementia (ADD) among older people. Patients with DLB present with not only cognitive impairment as do patients with ADD, but also various symptoms such as Parkinsonism, visual hallucinations, REM sleep behavioral disorder (RBD), cognitive fluctuation, and depressive mood from early stages onwards (1). Therefore, various factors contribute to deficits in activities of daily living (ADL) (2), and family caregivers experience a high burden (3–5) in cases of patients with DLB rather than those with ADD, although there is a report that shows no difference in caregiver burden between DLB and ADD (6).

Previous studies have documented that the high caregiver burden in DLB is affected by behavioral and psychological symptoms of dementia (BPSD) in addition to impaired ADL (4, 5). However, it is still unclear what kind of BPSD affects caregiver burden in DLB. The variety and interaction of symptoms in DLB may render it difficult to shed light on the relationship between caregiver burden and BPSD. Previous studies of ADD and mild cognitive impairment (MCI) revealed that BPSD consists of three factors of hyperactivity, and affective and psychotic symptoms, resulting from factor analysis of the 12-item Neuropsychiatric Inventory (NPI) (7, 8). Similar clusters of BPSD in DLB are considered to exist, but have not yet been confirmed.

The present study sought to verify the factors of BPSD in DLB using an exploratory factor analysis of NPI, and to evaluate the association between caregiver burden and each factor of BPSD in DLB.

## Methods

### Study Design

This study was carried out as a retrospective observational study without any intervention, and all participant's information was anonymized as unlinked data prior to analysis to prevent the identification of personal information. The authors assert that all procedures contributing to this work comply with the ethical standards of the relevant national and institutional committees on human experimentation and with the Helsinki Declaration of 1975, as revised in 2008. The study was approved by the Research Ethical Committee of the Osaka University Hospital (Suita, Japan).

### Participants

We retrospectively recruited patients diagnosed with probable DLB at the first diagnosis in our clinic from the database of neuropsychology clinics in the Department of Psychiatry at Osaka University Hospital from April 2009 to December 2019. The diagnosis of probable DLB was made according to the 2005 version of the international diagnostic criteria (9) until July 2017 and the 2017 version (10) after August 2017. Patients with Parkinson's disease dementia were excluded based on the 1-year rule according to both 2005 and 2017 criteria. We excluded patients without data related to NPI, caregiver burden, ADL, and Mini-Mental State Examination (MMSE) assessments.

### Clinical Feature Assessments

In our neuropsychological clinic, we assessed the physical condition, demographic data, medical history, and standard neuropsychological examination results. The patients also underwent routine laboratory tests and neuroimaging of the brain to exclude brain lesions, except for atrophy and physical condition, which might affect their symptoms.

General cognition and memory were examined using the MMSE (11), and logical memory of the Wechsler Memory Scale-Revised (WMS-R). ADL was evaluated using Lawton's Instrumental ADL and Physical Self-Maintenance Scale (IADL/PSMS) (12). The severity of dementia was assessed using the Clinical Dementia Rating (CDR) (13). The presence of the core clinical features of DLB was judged by specialists in geriatric psychiatry.

Neuropsychiatric symptoms and cognitive fluctuation were assessed using NPI-plus (14), which consists of the original 12 subitems of the NPI (15) with additional subitems for cognitive fluctuation. Neuropsychiatrists evaluated the neuropsychiatric symptoms of the patients using a semi-structured caregiver interview according to the NPI-plus manual. Caregivers were asked about the severity and frequency of 13 subitems in the 30 days before the interview. The frequency and severity of each subitem were classified as 0–3 and 0–4, respectively. Zero indicates absence of symptoms, and higher scores indicate that the symptom was worse or more frequent. The NPI composite score (frequency by severity) was calculated for each subitem (possible scores ranged from 0 to 12).

A Zarit Burden Interview (ZBI) was conducted to score caregiver burden (16). The ZBI consists of 22 self-rating questions for caregivers that examine the burden in the home care situation. Each item is rated from zero to four, and the total score ranges from zero to 88. Higher scores indicate a higher caregiver burden. ZBI has two subscales: personal strain (PS) factor indicates personal stressfulness of the experience, and role strain (RS) factor indicates the constraints on everyday life that occur as a result of being a caregiver.

### Statistical Analyses

The factor structure of NPI-plus was evaluated using exploratory factor analysis using the maximum likelihood method with varimax rotation. Before performing factor analysis, the suitability of the data was tested using the Kaiser–Meyer–Olkin (KMO) and Bartlett's tests. Factors with eigenvalues >1 were retained.

To assess the association between caregiver burden and symptoms, Spearman's rank correlation coefficients were calculated between total, PS, and RS scores of ZBI; factor scores resulting from the aforementioned factor analysis; age; MMSE score; the total score of five items scored in both males and females in IADL; and the total score of PSMS. To identify factors that independently influence caregiver burden, multiple regression analyses were conducted with the total, PS, and RS scores of ZBI as dependent variables and with factor scores resulting from the aforementioned factor analysis, age, sex, MMSE score, the total of five items in IADL, the total PSMS score, and the presence of Parkinsonism as independent variables.

These analyses were carried out in SPSS for Mac version 27.0 (IBM Corp., Armonk, NY, USA). The level of statistical significance was set at *p* < 0.05.

## Results

### Characteristics of Participants With DLB

**Figure 1:**
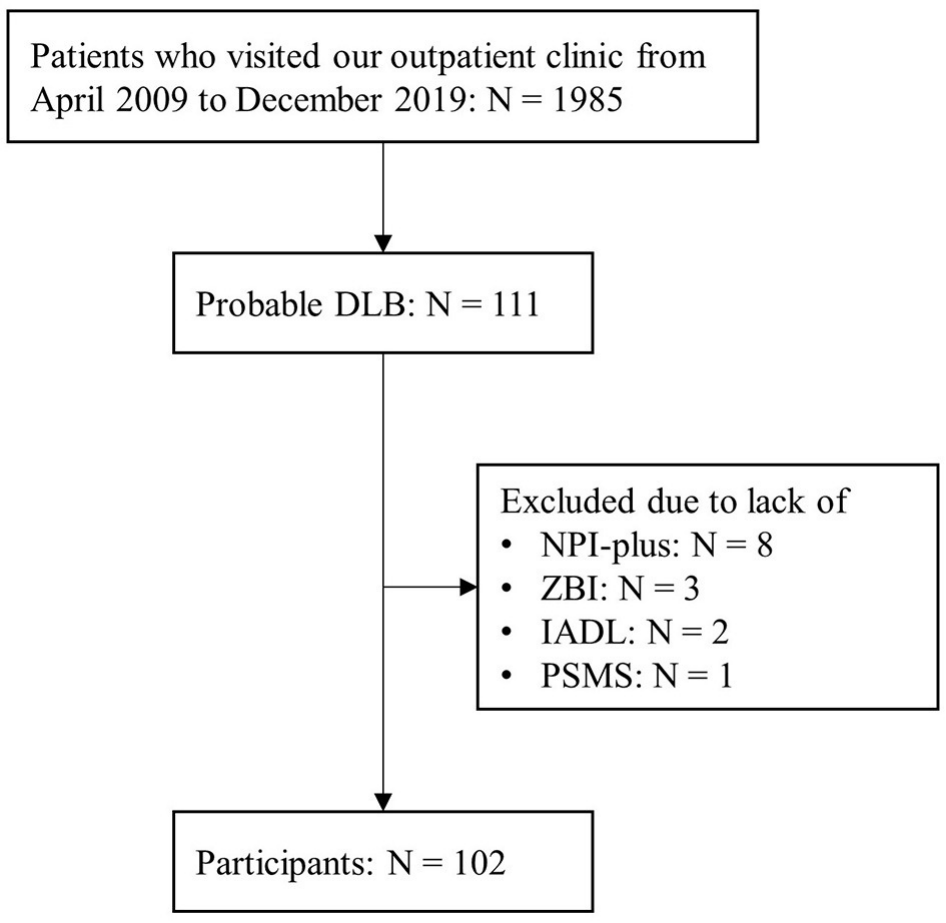
Participant selection.

Among the 1,985 patients who visited our outpatient clinic between April 2009 and December 2019, 111 were diagnosed with probable DLB. Nine patients were excluded due to lack of NPI-plus, ZBI, IADL/PSMS, or MMSE data, and 102 patients (mean [SD] age = 78.6 [5.6], 48 men and 54 women; mean [SD] MMSE = 20.2 [5.2]) were included in the present analysis (Figure 1, Table 1). Among them, 69 patients were diagnosed by the 2005 criteria, and 33 were diagnosed by the 2017 criteria. Among the core clinical features, cognitive fluctuation (68.6%) and visual hallucinations (72.6%) were prevalent.

**Table 1:** Study participant's characteristics.

Sex, male/female	48/54	Deficits in IADL	
Age	78.6 (5.6)	Telephone	5
MMSE	20.2 (5.2)	Shopping	56
WMS-R LM I^†^	7.3 (5.9)	Food preparation^††^	45
WMS-R LM II^†^	2.8 (4.0)	Housekeeping^††^	10
NPI-plus		Laundry^††^	18
Delusions	3.9 (4.1)	Transportation	3
Hallucinations	3.9 (3.8)	Medications	69
Agitation	1.0 (2.4)	Finance	23
Depression	1.6 (2.4)	Deficits in PSMS	
Anxiety	1.6 (2.9)	Toilet	25
Euphoria	0.0 (0.0)	Feeding	10
Apathy	4.8 (3.7)	Dressing	41
Disinhibition	0.4 (1.3)	Grooming	34
Irritability	1.2 (2.7)	Physical ambulation	49
Motor disturbances	1.0 (2.9)	Bathing	24
Nighttime behavior	3.1 (3.9)	ZBI	
Appetite	2.3 (3.7)	Total score	28.7 (18.5)
Total of NPI-12	24.6 (17.5)	Personal strain	16.0 (9.9)
Cognitive fluctuation	3.1 (3.3)	Role strain	7.0 (6.3)
Presence of core clinical		CDR, 0.5:1:2:3	38:39:22:3
features		Sum of box	6.3 (3.6)
Cognitive fluctuation	70	Prescription of ChEi	22
Visual hallucinations	74	Prescription of memantine	2
RBD	29		
Parkinsonism	53		

*Data represent the mean (SD) or number of participants*.

*MMSE, Mini-Mental State Examination; WMS-R, Wechsler Memory Scale Revised; LM, logical memory; NPI, Neuropsychiatric Inventory; RBD, REM sleep behavior disorder; IADL, Instrumental Activities of Daily Living; PSMS, physical self-maintenance scale; ZBI, Zarit Burden Interview; CDR, clinical dementia rating; ChEi, choline esterase inhibitor*.

† *Lack of data in two cases*,

†† *rated only in females*.

### Factor Structure of BPSD in DLB

Euphoria was not rated in any participants; therefore, we conducted an exploratory factor analysis for 12 items of NPI-plus, except for euphoria. The KMO measure was 0.666, indicating an appropriate sample size. Bartlett's test of sphericity revealed a statistical significance of ≤ 0.001, which confirms the goodness-of-fit of the model.

The exploratory factor analysis identified four factors (Table 2): the first factor consisted of hallucinations, delusions, and agitation related to “psychosis” (eigenvalue = 3.109); the second factor consisted of anxiety, appetite, depression, and apathy related to “affection” (eigenvalue = 1.686); the third factor consisted of nighttime behaviors and cognitive fluctuation related to “wakefulness” (eigenvalue = 1.334); the fourth factor consisted of motor disturbance and disinhibition related to “hyperactivity” (eigenvalue = 1.017). Irritability was not included in any factor because of the high cross-loading on the second and fourth factors.

**Table 2:** Factor loadings after Varimax rotation.

	**Factor 1: psychosis**	**Factor 2: affection**	**Factor 3: wakefulness**	**Factor 4: hyperactivity**
Hallucinations	**0.926**	−0.119	0.183	0.029
Delusions	**0.663**	0.084	0.069	0.094
Agitation	**0.301**	0.107	0.264	0.125
Anxiety	0.327	**0.809**	0.077	−0.227
Appetite	−0.023	**0.533**	0.199	0.051
Depression	−0.006	**0.438**	0.027	0.278
Apathy	−0.060	**0.337**	0.003	0.227
Nighttime behaviors	0.139	0.132	**0.980**	−0.049
Cognitive fluctuation	0.279	0.116	**0.339**	0.124
Motor disturbance	0.319	0.004	0.145	**0.583**
Disinhibition	0.008	0.097	−0.022	**0.507**
Irritability	0.272	0.363	0.255	0.368
Initial eigenvalues	3.109	1.686	1.334	1.017
Explained variance (%)	25.910	14.048	11.121	8.475
Cumulative variance (%)	25.910	39.958	51.079	59.554

*Bold indicates factor loading of symptom belonging to each factor*.

### Caregiver Burden and BPSD in DLB

Spearman's rank correlation coefficients showed significant positive correlations between the total score of ZBI and “psychosis” factor, as well as between total, PS, and RS scores of ZBI and “affection” factor (Table 3). There were also significant negative correlations between ZBI and MMSE, the total score of 5 items in IADL, and the total PSMS score.

**Table 3:** Spearman's rank correlation coefficients between caregiver burden and clinical symptoms.

	**ZBI total score**		**Personal strain**		**Role strain**	
	***r***	***p***	***r***	***p***	***r***	***p***
Factor 1: psychosis	0.206	0.038^*^	0.166	0.096	0.189	0.057
Factor 2: affection	0.219	0.027^*^	0.240	0.015^*^	0.219	0.027^*^
Factor 3: wakefulness	0.162	0.104	0.127	0.203	0.190	0.056
Factor 4: hyperactivity	−0.016	0.875	−0.020	0.843	0.009	0.928
Age	−0.023	0.817	−0.049	0.623	0.009	0.929
MMSE	−0.313	0.001^**^	−0.312	0.001^**^	−0.270	0.006^**^
5 items in IADL	−0.534	<0.001^**^	−0.506	<0.001^**^	−0.506	<0.001^**^
PSMS	−0.406	<0.001^**^	−0.377	<0.001^**^	−0.403	<0.001^**^

*ZBI, Zarit Burden Interview; MMSE, Mini-Mental State Examination; IADL, Instrumental Activities of Daily Living; PSMS, Physical Self-Maintenance Scale*.

* *p < 0.05*,

** *p < 0.01*.

In multiple regression analysis, the total score in ZBI was associated with “psychosis,” “affection,” and the total score of five items in IADL (Table 4). The PS score in ZBI was linked to “affection” and IADL. The RS score in ZBI was associated with “wakefulness” and IADL.

**Table 4:** Results of multiple regression analyses.

	**B**	**SE**	**95% CI**	***t***	***p***
**(A) Factors related to the total ZBI score**					
Intercept	56.64	24.33			
Factor 1: psychosis	3.66	1.74	0.20 to 7.12	2.10	0.039^*^
Factor 2: affection	3.91	1.85	0.24 to 7.58	2.12	0.037^*^
Factor 3: wakefulness	1.85	1.64	−1.41 to 5.12	1.13	0.262
Factor 4: hyperactivity	−0.34	2.06	−4.43 to 3.76	−0.16	0.871
Gender	−4.95	3.51	−11.92 to 2.03	−1.41	0.162
Age	0.09	0.30	−0.51 to 0.68	0.30	0.768
MMSE	0.00	0.34	−0.68 to 0.68	0.01	0.996
5 items in IADL	−5.73	1.93	−9.56 to −1.91	−2.98	0.004^**^
PSMS	−1.38	1.13	−3.63 to 0.87	−1.22	0.225
Presence of Parkinsonism	−3.16	3.31	−9.74 to 3.43	−0.95	0.343
**(B) Factors related to ZBI personal strain**					
Intercept	34.14	13.31			
Factor 1: psychosis	1.73	0.95	−0.16 to 3.63	1.82	0.072
Factor 2: affection	2.23	1.01	0.22 to 4.24	2.21	0.030^*^
Factor 3: wakefulness	0.53	0.90	−1.25 to 2.32	0.59	0.554
Factor 4: hyperactivity	−0.71	1.13	−2.95 to 1.53	−0.63	0.533
Gender	−1.76	1.92	−5.58 to 2.06	−0.92	0.362
Age	−0.01	0.16	−0.33 to 0.32	−0.04	0.967
MMSE	−0.03	0.19	−0.40 to 0.34	−0.17	0.869
5 items in IADL	−2.82	1.05	−4.91 to −0.72	−2.67	0.009^**^
PSMS	−0.85	0.62	−2.08 to 0.38	−1.37	0.173
Presence of Parkinsonism	−1.85	1.81	−5.45 to 1.75	−1.02	0.311
**(C) Factors related to ZBI role strain**					
Intercept	13.29	8.44			
Factor 1: psychosis	1.13	0.61	−0.07 to 2.34	1.87	0.064
Factor 2: affection	1.16	0.64	−0.11 to 2.44	1.81	0.073
Factor 3: wakefulness	1.14	0.57	0.01 to 2.28	2.01	0.048^*^
Factor 4: hyperactivity	0.29	0.72	−1.13 to 1.71	0.41	0.684
Gender	−2.31	1.22	−4.73 to 0.12	−1.89	0.062
Age	0.06	0.10	−0.14 to 0.27	0.60	0.548
MMSE	0.03	0.12	−0.20 to 0.27	0.27	0.787
5 items in IADL	−1.85	0.67	−3.18 to −0.52	−2.76	0.007^**^
PSMS	−0.35	0.39	−1.13 to 0.43	−0.90	0.369
Presence of Parkinsonism	−0.94	1.15	−3.22 to 1.35	−0.81	0.418

*ZBI, Zarit Burden Interview; MMSE, Mini-Mental State Examination; IADL, Instrumental Activities of Daily Living; PSMS, Physical Self-Maintenance Scale*.

* *p < 0.05*,

** *p < 0.01*.

## Discussion

This study sheds light on the characteristics of BPSD and the association between BPSD and caregiver burden in DLB. The factor analysis revealed four factors of BPSD in DLB, namely “psychosis,” “affection,” “wakefulness,” and “hyperactivity.” Caregiver burden was linked to a number of symptoms, and multivariate regression analyses revealed that each BPSD factor affected different aspects of caregiver burden.

The exploratory factor analysis revealed that BPSD was classified into four factors. Three of them, “psychosis,” “affection,” and “hyperactivity,” were reported in previous studies on ADD, MCI, and vascular cognitive impairment (7, 8, 17, 18) and were considered to be common factors of DLB and other dementia. The other “wakefulness” factor consisted of nighttime behaviors and cognitive fluctuation, which are specific symptoms in DLB compared with other types of dementia. We previously reported that increased limb movement during nighttime sleep, as measured by actigraphy, was related to cognitive fluctuation (19). The characteristics of cognitive fluctuation in DLB have been reported as disturbed arousal, such as excessive daytime sleepiness (20). As such, cognitive fluctuation is considered to be caused by sleep–wake disorder, and the “wakefulness” factor would be reasonable as a factor characteristic of DLB.

Alongside MMSE, IADL, and PSMS, the factors of “psychosis” and “affection” in BPSD were positively associated with caregiver burden in DLB. “Wakefulness” factor tended to be associated with the RS score in ZBI. These factors are frequently observed in DLB (10). These results are consistent with those of previous studies showing a relationship between BPSD and caregiver burden in DLB (3, 4). However, “hyperactivity” factor was not related to caregiver burden. In general, hyperactivity symptoms have been reported to affect caregiver burden in patients with dementia (21, 22), which is contrary to the present results. This may be influenced by the fact that motor disturbance and disinhibition in “hyperactivity” were less common in the current participants, which is consistent with the results of a previous multicenter study on BPSD (23). A systematic review showed that irritability, sleep disturbances, and anxiety were the most reported symptoms to impact caregivers, and that irritability, agitation, delusions, and apathy contribute the most to overall caregiver burden in patients with dementia (24). These symptoms were included in the “psychosis,” “affection,” and “wakefulness” factors, suggesting that the present results are consistent with the findings of this study. A study examining the impact of BPSD on caregiver distress in DLB and ADD reported that disinhibition, apathy, irritability, agitation, and aberrant motor behavior were important contributors to caregiver distress in ADD, whereas apathy, sleep disturbances, and aberrant motor behavior affected caregiver distress in DLB (25). Compared with ADD, “affection” and “wakefulness” factors may have more impact on caregiver burden in DLB.

Multiple regression analyses demonstrated that deficits in IADL were independently associated with all of the total, PS, and RS scores in ZBI after correcting various patient's conditions. The results agree with the relationship between caregiver burden and IADL reported in previous studies on dementia (21, 26) including DLB (3, 27). Multiple regression analyses also revealed that the “psychosis” factor was also associated with the total score in ZBI, and that the “affection” factor was independently associated with the total and PS scores in ZBI. A previous study examining the relationship between caregiver burden and symptoms in Lewy body dementia reported that IADL and non-motor symptoms were related to caregiver burden in DLB, whereas motor symptoms and IADL were related to caregiver burden in Parkinson's disease dementia (28). The previous and the present results are consistent in suggesting that non-motor symptoms, including BPSD, have a greater impact on the caregiver burden in DLB than motor symptoms. A previous validation study of ZBI reported that PS was related to BPSD, while RS was related to ADL deficits in impaired older people (29). We also reported that an improvement in BPSD was related to a decrease in PS in ZBI after shunt surgery in patients with iNPH (30). Therefore, these results replicate the association between BPSD and PS of ZBI in DLB. By contrast, the “wakefulness” factor was associated with the RS score in ZBI. As previously mentioned, the “wakefulness” factor is considered to be strongly related to daytime excessive sleepiness and to be more closely related to ADL than other BPSD. Therefore, this factor may be more related to RS than to PS.

In the present study, about 53% of the participants were females. Originally, it was often reported that males were more common in DLB in the 1990s (31–34). However, it has also been reported that females are more prevalent in DLB since 2000 (35–37). Gender difference in DLB may be debatable. In addition, it was reported that DLB patients with auditory hallucinations as their first symptom were more likely to be female (38). Since the patients with DLB in the present study were recruited from our outpatient clinic in Department of Psychiatry, it is possible that the number of patients with such psychiatric symptoms as their first symptoms was relatively high.

This study has several key strengths. First, we recruited over 100 patients with probable DLB, which is a sufficient number to perform a factor analysis on 12 variables and multivariate regression analyses. Second, we evaluated cognitive fluctuations in the same manner as the NPI. Therefore, we conducted a factor analysis with cognitive fluctuation in addition to various BPSDs and could reveal the “wakefulness” factor specific to DLB.

This study is also limited in a number of ways. First, this was a single-center, retrospective, cross-sectional study, which may have incurred a certain degree of bias. Second, we used two diagnostic criteria for probable DLB depending on when the patient visited our clinic. However, most current participants who were diagnosed until July 2017 also met the 2017 version of the diagnostic criteria for probable DLB. Third, we did not conduct confirmatory factor analysis because there were no previous studies that conducted a factor analysis of BPSD in DLB alone. Finally, some patients were prescribed choline esterase inhibitors and/or memantine, which might affect BPSD. However, the number of patients actually being prescribed these was relatively small. This may be due to the fact that many patients were undiagnosed in the early stages due to the nature of the facilities from which they were recruited. In fact, 38 of the participants were judged 0.5 in CDR, 39 were judged 1 in CDR, and more than 2/3 of the participants were very mild to mild stage of DLB. It should be noted that the results of this study are the findings in patients with these characteristics.

In conclusion, this study demonstrated that BPSD in DLB consists of four factors: “psychosis,” “affection,” “wakefulness,” and “hyperactivity.” Three factors were common to other dementia, and the “wakefulness” factor was considered to extract the characteristics of BPSD in DLB. In addition to IADL, “psychosis,” “affection,” and “wakefulness” factors of BPSD could affect caregiver burden. It is clinically noteworthy that factors of BPSD other than “hyperactivity,” which is generally reported to influence caregiver burden, may influence caregiver burden in DLB.

## Data Availability Statement

The raw data supporting the conclusions of this article will be made available by the authors, without undue reservation.

## Ethics Statement

The studies involving human participants were reviewed and approved by the Research Ethical Committee of the Osaka University Hospital (Suita, Japan). Written informed consent for participation was not required for this study in accordance with the national legislation and the institutional requirements.

## Author Contributions

HK designed the study and wrote the initial draft of the article. MI contributed to the interpretation of data and assisted in the preparation of the article. SS, YS, FK, DT, AK, TW, and KY have contributed to data collection, interpretation, and reviewed the article. All authors approved the article and agreed to be accountable for all aspects of the work in ensuring that questions related to the accuracy or integrity of any part of the work are appropriately investigated and resolved.

## Funding

This work was supported by JSPS KAKENHI under Grant No. T21K157300 and AMED under Grant No. JP21dk0207056.

## Conflict of Interest

HK, KY, and MI report grants and personal fees from Eisai Inc. The remaining authors declare that the research was conducted in the absence of any commercial or financial relationships that could be construed as a potential conflict of interest.

## Publisher's Note

All claims expressed in this article are solely those of the authors and do not necessarily represent those of their affiliated organizations, or those of the publisher, the editors and the reviewers. Any product that may be evaluated in this article, or claim that may be made by its manufacturer, is not guaranteed or endorsed by the publisher.
